# Sexually transmitted diseases knowledge assessment and associated factors among university students in the United Arab Emirates: a cross-sectional study

**DOI:** 10.3389/fpubh.2023.1284288

**Published:** 2023-11-23

**Authors:** Abdulla Alshemeili, Ahmed Alhammadi, Abdulrahman Alhammadi, Mohamed Al Ali, Eisa Saud Alameeri, Aminu S. Abdullahi, Bayan Abu-Hamada, Mohamud M. Sheek-Hussein, Rami H. Al-Rifai, Iffat Elbarazi

**Affiliations:** Institute of Public Health, College of Medicine and Health Sciences, United Arab Emirates University, Al Ain, United Arab Emirates

**Keywords:** STD, STI, knowledge, United Arab Emirates, students

## Abstract

**Background:**

Sexually transmitted diseases and infections (STDIs) remain a serious public health menace with over 350 million cases each year. Poor knowledge of STDIs has been identified as one of the bottlenecks in their control and prevention. Hence, assessment of knowledge, both general and domain-specific, is key to the prevention and control of these diseases. This study assessed the knowledge of STDIs and identified factors associated with STDI knowledge among university students in the United Arab Emirates (UAE).

**Methods:**

This is a cross-sectional study among 778 UAE University students across all colleges. An online data collection tool was used to collect data regarding the participants' demographics and their level of knowledge of STDIs across different domains including general STDI pathogens knowledge (8 items), signs and symptoms (9 items), mode of transmission (5 items), and prevention (5 items). Knowledge was presented both as absolute and percentage scores. Differences in STDI knowledge were statistically assessed using Mann-Whitney U and Chi-squared tests. Logistic regression models were further used to identify factors associated with STDI knowledge.

**Results:**

A total of 778 students participated in the study with a median age of 21 years (IQR = 19, 23). The overall median STDI knowledge score of the participants was 7 (out of 27), with some differences within STDI domains–signs & symptoms (1 out of 9), modes of transmission (2 out of 5), general STDI pathogens (2 out of 8), and prevention (1 out of 5). Higher STDI knowledge was significantly associated with being non-Emirati (OR = 1.85, 95% CI = 1.24–2.75), being married (OR = 2.89, 95% CI = 1.50–5.56), residing in emirates other than Abu Dhabi (OR = 1.61, 95% CI = 1.16–2.25), and being a student of health sciences (OR = 4.45, 95% CI = 3.07–6.45).

**Conclusion:**

In general, STDI knowledge was low among the students. Having good knowledge of STDIs is essential for their prevention and control. Therefore, there is a need for informed interventions to address the knowledge gap among students, youths, and the general population at large.

## 1 Introduction

Sexually Transmitted Diseases or Infections (STDIs) are passed from one individual to another through any kind of sexual contact. These diseases include a wide range of bacterial, viral, and fungal infections including, but not limited to, Chlamydia, Gonorrhea, Genital Herpes, Human Papilloma Virus (HPV), Syphilis, and Human Immunodeficiency Virus (HIV) (1).

STDIs impose significant public health concerns worldwide. According to the World Health Organization (WHO), more than one million STDIs are acquired every day, with an estimated 376 million new cases each year of four major Curable STDIs-chlamydia, gonorrhea, syphilis and trichomoniasis. In 2020, an estimated 374 million new infections with chlamydia (129 million), gonorrhea (82 million), syphilis (7.1 million) and trichomoniasis (156 million) were reported globally (2). While ~38 million people worldwide were living with HIV (3). The prevalence of HPV infection in sexually active women was estimated to be between 60 and 80% (4). Chronic hepatitis B, another acquired STDI, affected an estimated 296 million people worldwide and resulted in around 820,000 deaths in 2019, mainly due to cirrhosis and hepatocellular carcinoma (5, 6). Overall, STDIs can have profound consequences, including increasing the risk of HIV acquisition, mother-to-child transmission resulting in stillbirth, neonatal death, and congenital deformities, as well as causing Pelvic Inflammatory Disease (PID) and infertility in women (7).

Numerous studies, conducted worldwide, have shed light on the inadequate education and awareness regarding STDIs, being a significant global public health challenge according to the World Health Organization (7). Studies have revealed low knowledge among adolescents except for HIV in some countries (8), as others reported a good overall understanding of common STDIs, while specific diseases such as chlamydia were not well known (9). Other global studies highlighted significant knowledge gaps concerning STDI symptoms, complications, transmission, preventive methods, and other STDIs (9) and gaps in knowledge and awareness about associated symptoms and complications (8, 10–13).

In the Arab world, data about STDIs knowledge, attitude and awareness are limited. Previous studies in Saudi Arabia indicated a lack of awareness and poor knowledge of STDIs among adolescents, and recent research in the same country showed persistently low knowledge levels even after more than a decade (14).

In the United Arab Emirates (UAE), STDIs may pose a burden due to many reasons, especially among youths (15). Practices such as unprotected sex, multiple sexual partners, and engaging in high-risk sexual behaviors such as drug use may add to this burden (15). Additionally, social and cultural norms may discourage discussions about sexual health and limit access to information and resources on prevention and treatment (16). People living with STDIs may face social and cultural stigma that inhibits access to care and support and hence delays diagnosis and treatment (17). These same factors are behind the main reason for the dearth of epidemiological data on STDIs, thereby masking the burden of these diseases (17). There is notable limited research on STDIs knowledge and awareness among the population in the UAE, where sexual health and STDIs are considered taboo subjects, making people hesitant to seek medical care or disclose their sexual history due to cultural and social stigma (15, 18–20). There are only two published studies that explored STDIs knowledge and awareness in the UAE (21, 22). The most recent study, primarily focused on assessing knowledge and attitudes toward HIV/AIDS among university students, revealed significantly lower levels of knowledge among Emirati nationals compared to non-Emiratis (21).

This study assessed the knowledge of STDIs and explored factors associated with STDIs knowledge among university students in the UAE.

## 2 Methods

### 2.1 Study design, setting and sample size

A cross-sectional survey was carried out among students enrolled in the UAE University (UAEU). The UAEU is the first federal and the largest university in the UAE. The student population is mainly national local Emiratis, however, students in many colleges other than Engineering and Medical Colleges are composed of a variety of nationalities. Students in this university come from different emirates and are considered a good representation of the UAE youth population.

The optimal sample size for the study was estimated using a confidence interval of 95%, a margin of error of 5%, and an anticipated proportion of 50% (this gave us the maximum sample size as we did not have a precise reference estimate to use). This gave us a total sample of 377. Further, to address the issue arising from not using a simple random sampling technique, a design effect of 2 was used to multiply the estimated sample size making a final adequate sample size of 754. At the end, a total of 778 students responded to the survey, giving us a little higher sample size than what was estimated, and all were included in the analysis.

### 2.2 Study instrument and data collection

The survey tool was adopted from a previous study conducted in Saudi Arabia that explored STDI knowledge among university students and youths (23). The questionnaire was obtained from the original authors in both Arabic and English. The research team reviewed all questions and made sure that they were clear and culturally suitable. Then the survey was distributed to 20 students from the medical college to be reviewed and to pilot test its understandability. No changes were made to the original questionnaire except for the demographics.

The survey tool included two main sections assessing students' demographics and their knowledge of STDIs. The latter consisted of 27 knowledge-related questions distributed into four main domains–signs and symptoms, modes of transmission, prevention & protection, and general STDI pathogens knowledge. The survey link was distributed through an institutional office at the university to all university students (Undergraduates and Graduates) at all study levels and from all colleges twice at different time intervals. Hence, the data collection followed more of a convenience sampling, although the snowball sampling technique was also later employed to increase the response rate. University students above 18 years of age of all sexes and all colleges and study levels were eligible to answer the survey.

### 2.3 Ethics

Ethics approval was obtained from the Social Science Research Ethics Committee at the UAE University ERS_2022_8506. Participation in the study was voluntary. Information about the survey and the study team and contact were included at the beginning of the survey. Students were prompted to contact the study team and PI for any further information or to know the correct responses after the survey.

### 2.4 Data analysis

Participants' characteristics were summarized using frequencies and percentages, and median and interquartile range for categorical and quantitative characteristics, respectively. Regarding knowledge, each question or assessment statement was assigned a score of 1 if answered correctly and 0 if answered wrongly by the participants. Each participant's total knowledge score was then calculated by summing up scores from all the questions. Knowledge score was described as median and interquartile range owing to its distribution being skewed as tested both graphically and by the Shapiro-Wilk normality test. Further, the total knowledge score was categorized into “below median” and “above median” scores, using the overall participants' average score as cut off. This categorization of knowledge score was solely to facilitate comparison, and a similar approach was used previously (14). The score categories were summarized using frequencies and percentages.

Differences in the level of knowledge across participants' characteristics were explored using the Mann-Whitney U test and Kruskal-Wallis ANOVA (for continuous characteristics–i.e., age) and the Chi-squared test (for categorical characteristics). To identify factors associated with knowledge level and quantify the extent of the association, univariate and multivariable logistic regression models were used with the level of knowledge as the dependent variable. Only variables found to be significantly associated with the level of knowledge from the univariate analysis were included in the models, except “academic program” which was found to exhibit collinearity with “age.” All inferential statistics were performed at a significant level of 0.05. The Statistical Package for Social Sciences (version 28.0) was used for the analysis (IBM Corp. Released 2021. IBM SPSS Statistics for Windows, Version 28.0. Armonk, NY: IBM Corp).

## 3 Results

### 3.1 Participants' characteristics

A total of 778 students participated in the study with a median age of 21 years (IQR = 19, 23) (Table 1). The majority were female (66.6%), non-Emiratis (78.5%), unmarried (90.1%), and/or residing in the Emirate of Abu Dhabi (67.2%). Undergraduate students (86.1%) and those from the Faculty of Technology constituted the highest percentage of the respondents (34.2%), followed by students from the College of Health Sciences (25.6%).

**Table 1:** Participants' characteristics by STDI overall knowledge score (*N* = 778).

**Characteristics**	**All** ***n*** **(%)**	**Knowledge score Median (IQR)**	***P*-value**	**Knowledge level**		***P*-value**
				**Below median** ***n*** **(%)**	**Above median** ***n*** **(%)**	
**Age (years)**						
Median (IQR)	21 (19, 23)	–	–	21 (19, 23)	22 (19, 23)	0.004
**Gender**						
Female	518 (66.6)	7 (9.0)	0.338	267 (51.5)	251 (48.5)	0.362
Male	260 (33.4)	6 (10.0)		143 (55.0)	117 (45.0)	
**Nationality**						
UAE	611 (21.5)	6 (9.5)	<0.001	344 (56.3)	267 (43.7)	<0.001
Others	167 (78.5)	10 (10.0)		66 (39.5)	101 (60.5)	
**Marital status**						
Unmarried	701 (90.1)	6 (9.0)	<0.001	390 (55.6)	311 (44.4)	<0.001
Married	77 (9.9)	11 (10.0)		20 (26.0)	57 (74.0)	
**Emirate of residence**						
Abu Dhabi	523 (67.2)	6 (10.0)	0.007	290 (55.4)	233 (44.6)	0.028
Others	255 (32.8)	8 (10.0)		120 (47.1)	135 (52.9)	
**College**						
Health	199 (25.6)	13 (12.5)	<0.001	54 (27.1)	145 (72.9)	<0.001
Science	106 (13.6)	7 (8.0)		55 (51.9)	51 (48.1)	
Technology	266 (34.2)	5 (8.0)		171 (64.3)	95 (35.7)	
Others	206 (26.5)	6 (9.0)		129 (62.6)	77 (37.4)	
**Academic program**						
Undergraduate	669 (86.1)	6 (9.0)	<0.001	368 (55.0)	301 (45.0)	<0.001
Postgraduate	108 (13.9)	10 (12.0)		41 (38.0)	67 (62.0)	
**Unit of residence**						
Student dorm	159 (20.5)	7 (9.25)	0.583	84 (52.8)	75 (47.2)	0.957
Others	618 (79.5)	7 (9.0)		325 (52.6)	293 (47.4)	

IQR, interquartile range.

### 3.2 Knowledge of STDI

Overall, the median STDI knowledge score was 7.0 (IQR = 3.0, 12.0), which corresponds to an average percentage score of 25.9%. In terms of different STDI knowledge domains, the median domain-specific scores were 1.0 (IQR = 0.0, 3.0), 2.0 (IQR = 1.0, 3.0), 1.0 (0.0, 2.0), and 2.0 (IQR = 1.0, 5.0) for *signs & symptoms, modes of transmission, prevention*, and *general STDI pathogens*, respectively, which correspond to median percentage scores of 9.1, 33.3, 20.0, and 25.0% (Table 2). Additionally, a summary of responses to specific questions across various knowledge domains is provided in Table 3.

**Table 2:** Knowledge score by various domains of knowledge.

**Knowledge domain**	**Median (IQR), (Range)**	**Mean (±SD)**	**All correct (%)**	**None correct (%)**
Signs and symptoms (potential range: 0–9)	1.0 (0.0–3.0), (0.0–9.0)	2.1 (±2.3)	0	31.5
Mode of transmission (potential range: 0–5)	2.0 (1.0–3.0), (0.0–6.0)	1.9 (±1.4)	4.5	17.2
Prevention and protection (potential range: 0–5)	1.0 (0.0–2.0), (0.0–5.0)	1.2 (±1.4)	3.0	40.4
General STDI pathogens knowledge (potential range: 0–8)	2.0 (1.0–5.0), (0.0–8.0)	2.9 (±2.5)	7.1	20.2
Overall (potential range: 0–27)	7.0 (3.0–12.0), (0.0–27.0)	8.2 (±6.5)	0.1	7.3

IQR, Interquartile range; SD, Standard deviation.

**Table 3:** Summary of participants' responses to the STD knowledge assessment statements (*N* = 778).

**Statements**	**Correct answer**	
	***n***	**%**
**Signs and symptoms**		
Human Papillomavirus (HPV) can cause Genital Warts.	253	32.5
STDs can lead to health problems that are usually more serious for men than women.	245	31.5
If a person tests positive for HIV the test can tell how sick the person will become.	244	31.4
Soon after infection with HIV a person develops open sores on his or her genitals (penis or vagina).	160	20.6
A woman can look at her body and tell if she has Gonorrhea.	155	19.9
A woman can tell by the way her body feels if she has a Sexually Transmitted Disease.	150	19.3
A man can tell by the way his body feels if he has Hepatitis B.	145	18.6
Frequent urinary infections can cause Chlamydia.	143	18.4
A woman can tell that she has Chlamydia if she has a bad-smelling odor from her vagina.	136	17.5
**Mode of transmission**		
It is easier to get HIV if a person has another Sexually Transmitted Disease.	448	57.6
Having anal sex increases a person's risk of getting Hepatitis B.	315	40.5
A woman who has Genital Herpes can pass the infection to her baby during childbirth.	291	37.4
A man must have vaginal sex to get Genital Warts.	282	36.2
A person who has Genital Herpes must have open sores to give the infection to his or her sexual partner.	181	23.3
**Prevention and protection**		
There is a vaccine that can protect a person from getting Hepatitis B.	299	38.4
A man can protect himself from getting Genital Warts by washing his genitals after sex.	181	23.3
There is a vaccine that prevents a person from getting Chlamydia.	171	22.0
There is a vaccine available to prevent a person from getting Gonorrhea.	160	20.6
Using a natural skin (lambskin) condom can protect a person from getting HIV.	147	18.9
**General STD pathogens knowledge**		
The same virus causes all of the Sexually Transmitted Diseases.	471	60.5
There is a cure for Gonorrhea.	308	39.6
Human Papillomavirus (HPV) can lead to cancer in women.	278	35.7
Human Papillomavirus (HPV) is caused by the same virus that causes HIV.	275	35.3
There is a cure for Chlamydia.	261	33.5
Genital Herpes is caused by the same virus as HIV.	260	33.4
If a person had Gonorrhea in the past, he or she is immune (protected) from getting it again.	238	30.6
Human Papillomavirus (HPV) can cause HIV.	203	26.1

### 3.3 Factors associated with knowledge of STDI

The participants with a higher level of STDI knowledge were slightly, but significantly, older than those with a lower level of STDI knowledge (22 vs. 21 years; *P* = 0.004) (Table 1). Non-UAE nationals had a significantly higher prevalence of high knowledge than the UAE nationals (60.5 vs. 43.7%; *P* < 0.001). Prevalence of high knowledge was also higher among married compared to unmarried participants (74 vs. 44%; *P* < 0.001). Participants residing outside Abu Dhabi Emirates had a significantly higher prevalence of high STDI knowledge (52.9 vs. 44.6%; *P* = 0.028). Significantly (*P* < 0.001) highest prevalence of high STDI knowledge was also seen among health students (72.9%) followed by science students (48.1%) as technology students got the least (35.7%). Postgraduate students had a significantly higher prevalence of high STDI knowledge than undergraduate students (62 vs. 45%; *P* < 0.001). On the other hand, gender (*P* = 0.362), and students' unit of residence (*P* = 0.957) were not associated with level of knowledge.

Moreover, across all domains of STDI knowledge, other nationalities had significantly higher knowledge scores than the UAE nationals; Married participants had significantly higher knowledge scores than the unmarried; Finally, students from Health Science College recorded significantly higher knowledge scores than those from other colleges (Table 4).

**Table 4:** Domain-specific knowledge scores by nationality, marital status, and college.

**Characteristics**	**Prevention**	***P*-value**	**Transmission mode**	***P*-value**	**General pathogens**	***P*-value**
**Nationality**						
UAE	1.0 (0.0–2.0)	**0.001** ^a^	2.0 (1.0–3.0)	**0.005** ^b^	2.0 (1.0–5.0)	**<0.001**
Others	1.0 (0.0–3.0)		2.0 (1.0–3.0)		4.0 (1.0–6.0)	
**Marital status**						
Unmarried	1.0 (0.0–2.0)	**<0.001**	2.0 (1.0–3.0)	**<0.001** ^c^	2.0 (1.0–5.0)	**<0.001**
Married	2.0 (1.0–3.0)		2.0 (1.5–4.0)		4.0 (2.0–6.0)	
**College**						
Health	2.0 (1.0–3.0)	**<0.001**	2.0 (1.0–4.0)	**<0.001**	5.0 (2.0–7.0)	**<0.001**
Science	1.0 (0.0–2.0)		2.0 (1.0–3.0)		2.5 (1.0–4.0)	
Technology	0.0 (0.0–1.0)		1.0 (1.0–2.0)		1.5 (0.0–4.0)	
Others	1.0 (0.0–1.0)		2.0 (1.0–3.0)		2.0 (0.8–4.0)	

Mann-Whitney U test was used to test differences in knowledge scores across nationality and marital status, while Kruskal-Wallis' ANOVA was used to test differences in knowledge scores across college categories.

a While the median scores were equal, the mean rank score for other nationalities (437.4) was higher than for UAE nationals (376.4).

b While the median scores were equal, the mean rank score for other nationalities (431.6) was higher than for UAE nationals (378.0).

c While the median scores were equal, the mean rank score for married (474.8) was higher than for unmarried (380.1). Bold values indicate the statistical significance.

In a multivariable logistic regression analysis to investigate factors associated with level of knowledge after adjusting for the effect of possible confounders (Table 5), significantly higher odds of higher knowledge were found among other nationalities compared to UAE nationals (OR = 1.85, 95% CI = 1.24–2.75), married compared to unmarried (OR = 2.89, 95% CI = 1.50–5.56), those residing in other Emirates compared to those residing in Abu Dhabi Emirate (OR = 1.61, 95% CI = 1.16–2.25), and among those from the College of Health Sciences compared to others (OR = 4.45, 95% CI = 3.07–6.45).

**Table 5:** Univariate and multivariable logistic regression model for the association between STDI knowledge and participants' characteristics.

**Characteristics**	**Crude OR (95% CI)**	**Adjusted OR (95% CI)**
Age (years)	1.06 (1.03, 1.09)^*^	1.01 (0.97, 1.05)
**Nationality**		
UAE	1	1
Other nationalities	1.97 (1.39, 2.80)^*^	1.85 (1.24, 2.75)^*^
**Marital status**		
Unmarried	1	1
Married	3.57 (2.10, 6.08)^*^	2.89 (1.50, 5.56)^*^
**Emirate of residence**		
Abu Dhabi	1	
Other emirates	1.40 (1.04, 1.89)^*^	1.61 (1.16, 2.25)^*^
**College**		
Other colleges	1	1
Health	4.28 (3.00, 6.09)^*^	4.45 (3.07, 6.45)^*^

OR, odds ratio; CI, confidence interval;

* P < 0.05; Adjustment was made for all the variables in the model.

## 4 Discussion

The present study assessed the knowledge of STDIs, and identified factors associated with STDI knowledge among university students in the UAE.

Overall, the study found that the average knowledge score among the participants was 25.9%. This result is consistent with previous studies that reported low levels of STDIs knowledge among young adults in Europe, Saudi Arabia and Vietnam (8, 23, 24). The study found that age, nationality, marital status, emirate of residence, and college were significantly associated with the level of STDI knowledge. Specifically, participants who were slightly older, non-UAE nationals, married, residing outside Abu Dhabi Emirates, and those from the College of Health Sciences had higher odds of demonstrating high knowledge of STDI. Gender and the students' unit of residence were not associated with the level of knowledge. Also, the study found that nationality, marital status, emirate of residence, and college were significant predictors of the level of STDI knowledge. These findings are in line with previous studies from Saudi Arabia, Bangladesh, and Greece that have identified similar factors associated with higher levels of STDI knowledge (14, 25, 26). Married participants had higher STDI knowledge than the unmarried which can be explained by the knowledge they were exposed to through the premarital screening tests which include screening for infectious diseases (27). Higher STDI knowledge among health sciences students may be attributed to the nature of the health sciences educational curriculum that equips the students with adequate knowledge of infectious diseases including STIs. Expatriates undergo a compulsory medical screening as a condition for residence visa, and this screening includes some infectious diseases, which may explain the higher knowledge among the expatriates. Lower knowledge among students living in Abu Dhabi compared to other emirates may be difficult to explain and perhaps requires some further investigation, however, a similar study from Bangladesh reported significant difference in STDI knowledge across different geographic locations (25).

In the contrary, a study conducted among inmates of women shelter homes in Malaysia did not find demographic characteristics including age and socio-economic status to be significantly associated with STDIs knowledge (28). This disagreement with our findings could be explained by the difference in study population as their study was among women inmates.

Studies showed that university students have a basic understanding of STDIs, their transmission, and prevention methods. However, many lack in-depth knowledge about specific STDIs and their associated risks. In a study conducted among university students in Nigeria, 63.5% of the respondents had a good understanding of STDIs, while 36.5% had poor knowledge of STDIs. Similarly, a study conducted in Georgia, United States found that while most of the students had a basic understanding of STDs, only a few had comprehensive knowledge about the associated risks and long-term effects of STDIs (29). Attitudes toward STDIs among university students vary depending on the culture, beliefs, and social norms of the community. Stigma, embarrassment, and fear of judgment are common barriers to discussing STDIs and seeking medical attention. In a study conducted among university students in India, many respondents perceived STDIs as a sign of promiscuity and believed that they could not contract STDIs due to their clean and healthy lifestyle (30). Similarly, a study conducted among university students in Nigeria found that many students were reluctant to seek medical attention for STDIs due to the stigma attached to the disease (31).

Prevention methods for STDIs among university students include abstinence, consistent condom use, and regular screening. Studies showed that most university students are aware of these prevention methods but do not consistently practice them. In a study conducted among university students in Canada, only 32% of the respondents reported consistent condom use, while 19% reported engaging in unprotected sex (32). Similarly, a study conducted among university students in Nigeria found that many students were unaware of the availability of STDI screening services on campus and the importance of regular screening (33). Prevention and treatment of STDIs in the UAE are generally in line with international standards. The UAE Ministry of Health and Prevention provides guidance and resources on prevention, testing, and treatment of STDs.

Interestingly, the study found that being slightly older was associated with higher levels of STDI knowledge, which contradicts some previous studies that have found no association between age and STDI knowledge (26). It is possible that older participants in this study had more exposure to sexual health education and/or more sexual experiences, leading to greater knowledge about STDIs.

The study found that the participants displayed similar levels of low levels of knowledge in the general pathogens' knowledge, signs and symptoms, mode of transmission, and prevention methods which highlight those educational efforts should cover all these aspects of STDI awareness and literacy.

Studies reported low levels of awareness and knowledge of sexually transmitted diseases, among adolescents and youths in general (9). To sum up, university students possess fundamental knowledge regarding STDIs, how they spread, and ways to prevent them. However, their understanding of STDIs and the risks associated with them may not be comprehensive. The attitude of university students toward STDIs is shaped by cultural beliefs and social norms. Talking about and seeking medical assistance for STDIs are often hindered by stigma, embarrassment, and the fear of being judged (17).

To augment the problem of lack of awareness and access, the COVID-19 pandemic has impacted many aspects of healthcare, including the diagnosis, treatment, and prevention of STDIs. Many healthcare resources have been redirected toward managing the pandemic, reducing testing and screening for STDIs and access to clinics and other healthcare facilities providing these services.

The COVID-19 pandemic has also led to disruptions in the provision of treatment for STDIs (34). The distruption might have also led to decreased STI prevention service provision due to social isolation and the transfer of health workers to other facilities. Nonetheless, the COVID-19 impact was also reported to be positive as it has influenced practices such as a decrease in sexual activity or changes in sexual partners. However, other people have reported engaging in riskier sexual behaviors, such as having sex without a condom or engaging in group sex. People who are infected with COVID-19 may also be at increased risk of contracting STDIs or experiencing complications related to existing STDIs. This is because COVID-19 can weaken the immune system, making it more difficult to fight off infections. STDIs are a major public health concern worldwide, particularly among young adults. University students are at high risk of contracting STDIs due to their active sex life and potential engagement in risky sexual behavior (34).

Overall, the study provides valuable insights into the level of STDI knowledge among university students in a specific context, as well as factors associated with higher levels of knowledge. However, further research is needed to better understand the factors that contribute to low levels of STDI knowledge among university students and to develop effective educational interventions to improve knowledge and prevent the spread of STDIs.

The results of this study highlight the need for increased sexual health education among young adults, particularly those who are unmarried, UAE nationals, and/or technology students. Health promotion strategies targeting these groups may help to increase knowledge of STDIs and reduce the spread of these infections. Given that university students are young adults, targeted awareness campaigns are necessary to provide them with essential knowledge about STDIs, their causes, risk factors, transmission modes, and prevention methods (10, 17). Stigma, embarrassment, and the fear of judgment often hinder university students from seeking medical assistance for STDIs. Studies investigating the knowledge and attitudes of this vulnerable group are crucial in informing decision-makers about the need for targeted campaigns. Additionally, future research should explore the effectiveness of different educational interventions for improving knowledge of STDIs among young adults.

It is important to note that the study has several limitations that may affect the generalizability of the findings. For example, the study was conducted in a specific geographic location, and the sample primarily consisted of undergraduate students. Therefore, caution should be exercised when generalizing the findings to other populations or settings. Also, the nature of the study being a cross-sectional study may introduce recall and knowledge bias as people may search for certain answers. We believe such a bias is for the benefit of the participants as this may help in indirectly improving people's knowledge. The strengths of this include the issue of a survey that was used before in Saudi Arabia which has a close cultural and social resemblance to the UAE population. At the same time, this study will help break the taboo in discussing public health issues that may endure certain stigma and cultural unacceptance.

## 5 Conclusions

In conclusion, our study population demonstrated a basic understanding of STDIs, their transmission, and prevention methods. Higher knowledge of STDIs was significantly associated with non-UAE nationality, being married, residing in other emirates than Abu Dhabi, and being a health science student. However, many lacked in-depth knowledge about specific STDIs and their associated risks. Attitudes toward STDIs among university students are influenced by cultural beliefs and social norms. Stigma, embarrassment, and fear of judgment are common barriers to discussing STDIs and seeking medical attention. It is important to develop targeted interventions to address these gaps in knowledge among university students.

## Data availability statement

The raw data supporting the conclusions of this article will be made available by the authors, without undue reservation.

## Ethics statement

The study was approved by Social Science Research Ethics Committee at the UAE University ERS_2022_8506. The study was conducted in accordance with the local legislation and institutional requirements. The participants provided their written informed consent to participate in this study.

## Author contributions

AAl: Conceptualization, Writing—original draft, Methodology, Project administration. AhA: Conceptualization, Investigation, Writing—original draft. AbA: Conceptualization, Investigation, Writing—original draft. MA: Conceptualization, Investigation, Writing—original draft. EA: Conceptualization, Investigation, Writing—original draft. ASA: Data curation, Formal analysis, Writing—original draft, Writing—review & editing. BA-H: Investigation, Project administration, Writing—original draft. MS-H: Conceptualization, Project administration, Writing—review & editing, Methodology. RA-R: Writing—review & editing, Conceptualization, Methodology. IE: Conceptualization, Project administration, Writing—original draft, Writing—review & editing, Methodology, Supervision.

## References

[1] CDC. Sexually Transmitted Diseases (STDs). (2023). Available online at: https://www.cdc.gov/std/general/default.htm#:~:text=Sexually%20transmitted%20diseases%20(STDs)%2C%2C%20oral%2C%20and%20anal%20sex (accessed June 05, 2023).

[2] WHO. Sexually Transmitted Infections (STIs). (2022). Available online at: https://www.who.int/news-room/fact-sheets/detail/sexually-transmitted-infections-(stis) (accessed June 05, 2023).

[3] UNAIDS. Global HIV & AIDS statistics — Fact sheet. (2022). Available online at: https://www.unaids.org/en/resources/fact-sheet (accessed June 05, 2023).

[4] Soheili M Keyvani H Soheili M Nasseri S. Human papilloma virus: a review study of epidemiology, carcinogenesis, diagnostic methods, and treatment of all HPV-related cancers. Med J Islam Repub Iran. (2021) 35:65. 10.47176/mjiri.35.65 34277502 PMC8278030

[5] WHO. Global Progress Report on HIV, Viral Hepatitis and Sexually Transmitted Infections. Geneva: WHO (2021).

[6] de Martel C Georges D Bray F Ferlay J Clifford GM. Global burden of cancer attributable to infections in 2018: a worldwide incidence analysis. Lancet Glob Health. (2020) 8:e180–e90. 10.1016/S2214-109X(19)30488-7 31862245

[7] WHO. Global Health Sector Strategy on Sexually Transmitted Infections 2016–2021. (2016). Available online at: https://apps.who.int/iris/bitstream/handle/10665/246296/WHO-RHR-16.09-eng.pdf (accessed June 02, 2023).

[8] Samkange-Zeeb FN Spallek L Zeeb H. Awareness and knowledge of sexually transmitted diseases (STDs) among school-going adolescents in Europe: a systematic review of published literature. BMC Public Health. (2011) 11:1–12. 10.1186/1471-2458-11-727 21943100 PMC3189891

[9] Obiechina NJ Diwe K Ikpeze O. Knowledge, awareness and perception of sexually transmitted diseases (STDs) among Nigerian adolescent girls. J Obstet Gynaecol. (2002) 22:302–5. 10.1080/01443610220130634 12521506

[10] Anwar M Sulaiman SAS Ahmadi K Khan TM. Awareness of school students on sexually transmitted infections (STIs) and their sexual behavior: a cross-sectional study conducted in Pulau Pinang, Malaysia. BMC Public Health. (2010) 10:1–6. 10.1186/1471-2458-10-47 20113511 PMC2824738

[11] Mansor N Ahmad N Rahman HA. Determinants of knowledge on sexually transmitted infections among students in public higher education institutions in Melaka state, Malaysia. PLoS ONE. (2020) 15:e0240842. 10.1371/journal.pone.0240842 33119620 PMC7595423

[12] Koray MH Adomah-Afari A Punguyire D Naawa A. Knowledge of sexually transmitted infections among senior high school adolescents in the Wa Municipality of Ghana. Glob Health J. (2022) 6:95–101. 10.1016/j.glohj.2022.04.002

[13] Subbarao NT Akhilesh A. Knowledge and attitude about sexually transmitted infections other than HIV among college students. Indian J Sex Transm Dis AIDS. (2017) 38:10. 10.4103/0253-7184.196888 28442798 PMC5389207

[14] Albanghali MA Othman BA. A cross-sectional study on the knowledge of sexually transmitted diseases among young adults living in albaha, Saudi Arabia. Int J Environ Res Public Health. (2020) 17:1872. 10.3390/ijerph17061872 32183110 PMC7142563

[15] Eng TR Butler WT. Factors That Contribute to the Hidden Epidemic. The Hidden Epidemic: Confronting Sexually Transmitted Diseases. Washington, DC: National Academies Press (1997). 25165803

[16] Setayesh H Roudi-Fahimi F El Feki S Ashford LS. HIV and AIDS in the Middle East and North Africa. Washington, DC: Population Reference Bureau (2014).

[17] Malek AM Chang C-CH Clark DB Cook RL. Delay in seeking care for sexually transmitted diseases in young men and women attending a public STD clinic. Open AIDS J. (2013) 7:7–13. 10.2174/1874613620130614002 24078858 PMC3785038

[18] Barss P Grivna M Ganczak M Bernsen R Al-Maskari F El Agab H. Effects of a rapid peer-based HIV/AIDS educational intervention on knowledge and attitudes of high school students in a high-income Arab country. JAIDS J Acquir Immune Defic Syndr. (2009) 52:86–98. 10.1097/QAI.0b013e31819c153f 19590431

[19] Zaidi MA Griffiths R Newson-Smith M Levack W. Impact of stigma, culture and law on healthcare providers after occupational exposure to HIV and hepatitis C. Cult Health Sex. (2012) 14:379–91. 10.1080/13691058.2011.646304 22214403

[20] Bos AE Schaalma HP Pryor JB. Reducing AIDS-related stigma in developing countries: the importance of theory-and evidence-based interventions. Psychol Health Med. (2008) 13:450–60. 10.1080/13548500701687171 18825583

[21] Haroun D El Saleh O Wood L Mechli R Al Marzouqi N Anouti S. Assessing knowledge of, and attitudes to, HIV/AIDS among university students in the United Arab Emirates. PLoS ONE. (2016) 11:e0149920. 10.1371/journal.pone.0149920 26913902 PMC4767799

[22] Ortashi O Raheel H Shalal M Osman N. Awareness and knowledge about human papillomavirus infection and vaccination among women in UAE. Asian Pac J Cancer Prev. (2013) 14:6077–80. 10.7314/APJCP.2013.14.10.6077 24289628

[23] El-Tholoth HS Alqahtani FD Aljabri AA Alfaryan KH Alharbi F Alhowaimil AA. Knowledge and attitude about sexually transmitted diseases among youth in Saudi Arabia. Urol Ann. (2018) 10:198. 10.4103/UA.UA_14_17 29719334 PMC5907331

[24] Nguyen SH Dang AK Vu GT Nguyen CT Le THT Truong NT. Lack of knowledge about sexually transmitted diseases (STDs): Implications for STDs prevention and care among dermatology patients in an urban city in Vietnam. Int J Environ Res Public Health. (2019) 16:1080. 10.3390/ijerph16061080 30917565 PMC6466097

[25] Hossain M Mani KK Sidik SM Shahar HK Islam R. Knowledge and awareness about STDs among women in Bangladesh. BMC Public Health. (2014) 14:1–7. 10.1186/1471-2458-14-775 25081860 PMC4246425

[26] Voyiatzaki C Venetikou MS Papageorgiou E Anthouli-Anagnostopoulou F Simitzis P Chaniotis DI. Awareness, knowledge and risky behaviors of sexually transmitted diseases among young people in Greece. Int J Environ Res Public Health. (2021) 18:10022. 10.3390/ijerph181910022 34639324 PMC8508576

[27] Premarital Counselling and Screening. (2023). Available online at: https://u.ae/en/information-and-services/social-affairs/marriage/premarital-counselling-and-screening#:~:text=Premarital%20examination%20is%20a%20requirementinfectious%20diseases%20and%20genetic%20diseases (accessed October 24, 2023).

[28] Zin NM Ishak I Manoharan K. Knowledge, attitude and practice towards sexually transmitted diseases amongst the inmates of women shelters homes at Klang Valley. BMC Public Health. (2019) 19:1–7. 10.1186/s12889-019-6863-5 31196029 PMC6565535

[29] Shannon CL Klausner JD. The growing epidemic of sexually transmitted infections in adolescents: a neglected population. Curr Opin Pediatr. (2018) 30:137. 10.1097/MOP.0000000000000578 29315111 PMC5856484

[30] King KA Vidourek RA Singh A. Condoms, sex, and sexually transmitted diseases: exploring sexual health issues among Asian-Indian college students. Sex Cult. (2014) 18:649–63. 10.1007/s12119-013-9214-1

[31] Nzoputam C Adam VY Nzoputam O. Knowledge, prevalence and factors associated with sexually transmitted diseases among female students of a Federal University in Southern Nigeria. Venereology. (2022) 1:81–97. 10.3390/venereology1010006

[32] Milhausen RR McKay A Graham CA Crosby RA Yarber WL Sanders SA. Prevalence and predictors of condom use in a national sample of Canadian university students. Can J Hum Sex. (2013) 22:142–51. 10.3138/cjhs.2316

[33] Osanyin GE Ogunyemi DO Oluwole EO Oyekanmi OD. Knowledge, attitude and preventive practices of sexually transmitted infections among unmarried youths in an urban community in Lagos State, Nigeria. Afr J Prim Health Care Fam Med. (2020) 12:1–7. 10.4102/phcfm.v12i1.2221 32370529 PMC7203189

[34] CDC. Impact of COVID-19 on STDs. Available online at: https://www.cdc.gov/std/statistics/2021/impact.htm#:~:text=The%20COVID%2D19%20pandemic%20significantlyof%20the%20pandemic%20on%20STDs (accessed April 11, 2023).

